# Antimicrobial Activity of Drimanic Sesquiterpene Compounds from *Drimys winteri* against Multiresistant Microorganisms

**DOI:** 10.3390/molecules29122844

**Published:** 2024-06-14

**Authors:** Iván Montenegro, Rolando Pazmiño, Ileana Araque, Alejandro Madrid, Ximena Besoain, Enrique Werner, Luis Espinoza-Catalán, Andrés F. Olea, Claudio Parra, Valentina Navarrete Molina, Patricio Godoy, Yusser Olguín, Mauricio A. Cuellar

**Affiliations:** 1 Núcleo Milenio Bioproductos, Genómica Y Microbiología Ambiental—Biogem, Center of Interdisciplinary Biomedical and Engineering Research for Health (MEDING), Escuela de Obstetricia y Puericultura, Facultad de Medicina, Universidad de Valparaíso, Angamos 655, Reñaca, Viña del Mar 2520000, Chile; molinava.1998@gmail.com; 2 Centro de Investigación, Desarrollo e Innovación de Productos Bioactivos (CINBIO), Facultad de Farmacia, Escuela de Química y Farmacia, Universidad de Valparaíso, Av. Gran Bretaña 1093, Valparaíso 2340000, Chile; rapazmino@mail.com (R.P.); ileana.araque@postgrado.uv.cl (I.A.); 3 Laboratorio de Productos Naturales y Síntesis Orgánica (LPNSO), Departamento de Ciencias y Geografía, Facultad de Ciencias Naturales y Exactas, Universidad de Playa Ancha, Avda. Leopoldo Carvallo 270, Playa Ancha, Valparaíso 2340000, Chile; alejandro.madrid@upla.cl; 4 Escuela de Agronomía, Pontificia Universidad Católica de Valparaíso, Quillota, San Francisco s/n La Palma, Quillota 2260000, Chile; ximena.besoain@pucv.cl; 5 Departamento de Ciencias Básicas, Universidad del Bio-Bio Campus Chillán, Avda. Andrés Bello 720, Casilla 447, Chillán 3800708, Chile; ewerner@ubiobio.cl; 6 Departamento de Química, Universidad Técnica Federico Santa María, Avenida España 1680, Valparaíso 2340000, Chile; luis.espinozac@usm.cl; 7 Grupo QBAB, Instituto de Ciencias Aplicadas, Facultad de Ingeniería, Universidad Autónoma de Chile, El Llano Subercaseauux 2801, Santiago 8900000, Chile; andres.olea@uautonoma.cl; 8 Departamento de Química Orgánica, Facultad de Ciencias Químicas, Universidad de Concepción, Edmundo Larenas 129, Concepción 4070371, Chile; cparra@udec.cl; 9 Facultad de Medicina, Instituto de Microbiología Clínica, Universidad Austral de Chile, Los Laureles s/n, Isla Teja, Valdivia 5090000, Chile; patricio.godoy@uach.cl; 10 Departamento de Química y Medio Ambiente, Universidad Técnica Federico Santa María, Avenida España 1680, Valparaíso 2390123, Chile; yusser.olguin@usm.cl; 11 Centro Científico y Tecnológico de Valparaíso (CCTVal), Universidad Técnica Federico Santa María, Avenida España 1680, Valparaíso 2390123, Chile; 12 Centro de Biotecnología, Universidad Técnica Federico Santa María, Avenida España 1680, Valparaíso 2390123, Chile

**Keywords:** *Drimys winteri*, drimane sesquiterpenes, polygodial, antimicrobial activity

## Abstract

In this work, a group of ten sesquiterpene drimanes, including polygodial (**1**), isopolygodial (**2**), and drimenol (**3**) obtained from the bark of *Drimys winteri* F. and seven synthetic derivatives, were tested in vitro against a unique panel of bacteria, fungi, and oomycetes with standardized procedures against bacterial strains *K. pneumoniae*, *S. tiphy*, *E. avium*, and *E. coli.* The minimum inhibitory concentrations and bactericidal activities were evaluated using standardized protocols. Polygodial (**1**) was the most active compound, with MBC 8 μg/mL and MIC 16 μg/mL in *E. avium*; MBC 16 μg/mL and MIC 32 μg/mL in *K. pneumoniae*; MBC 64 μg/mL and MIC 64 μg/mL in *S. typhi*; and MBC 8 μg/mL and MIC 16 μg/mL and MBC 32 μg/mL and MIC 64 μg/mL in *E. coli*, respectively. The observed high potency could be attributed to the presence of an aldehyde group at the C8–C9 position. The antifungal activity of **1** from different microbial isolates has been evaluated. The results show that polygodial affects the growth of normal isolates and against filamentous fungi and oomycetes with MFC values ranging from 8 to 64 μg/mL. Sesquiterpene drimanes isolated from this plant have shown interesting antimicrobial properties.

## 1. Introduction

*Drimys winteri* J.R. Forster et G. Forster (Winteraceae) is a native tree in southern Chile and Argentina, commonly found in humid and even marshy lands [1]. The composition of *D. winteri* includes the presence of sesquiterpene- and lactone-type drimanes including polygodial (**1**), isopolygodial (**2**), and drimenol (**3**) [2]. In addition, many derivatives have been synthesized from these structures, offering biological activities such as antibacterial, antifungal, and larvicide [2,3,4]. The search for new antibacterial agents has led to the exploration of various natural and synthetic sources, intending to identify, develop, and discover new drugs [5]. Terpenes are the most numerous and structurally diverse class of natural products derived from the mevalonic acid (MVA) pathway. These compounds are characterized by different carbon skeletons, which comprise a variety of isoprene structural units [4,6,7]. Recent studies reported that 75% of the enhancers of antibacterial drugs belong to terpenes [8].

Drimanic compounds are sesquiterpenes with bicyclic farnesane-type skeletons, showing several significant bioactivities, such as antimicrobial [9], algaecide [10], antifouling [11], cytotoxic [12,13], and antifeedant activities [14]. The drimanic compounds possess antifungal activity [3,15]. Polygodial is the most widely occurring sesquiterpene dialdehyde, which has been reported worldwide in flowering plants, ferns, fungi, and marine mollusks [16]. Derita et al. (2013) studied (among other drimane sesquiterpenes) the +active compound **1** and found that the drimanic compound biological activity is likely due to the electronic properties around the Δ7,8 double bond. Active compounds had a large, positive differential zone in the molecular electrostatic potentials (MEPs) [15], which is probably responsible for its high antipathogenic activity. Anke also reported on the antibacterial activity of drimanic compound **1** against Gram-negative and Gram-positive bacteria at minimal inhibitory concentrations of 2–20 µg/mL [17]. Kubo also reported that compound **1** exhibited moderate antibacterial activity against Gram-positive bacteria such as *Bacillus subtilis* and *Staphylococcus aureus*, with minimum bactericidal concentration (MBC) values of 100 µg/mL; as well as against Gram-negative bacteria such as *Salmonella choleraesuis* and *Escherichia coli*, with MBC values of 50 µg/mL and 100 µg/mL, respectively [18]. Indeed, compound **1** inhibits the growth of phytopathogen bacteria *Ralstonia solanacearum* species complex (RSSC) with MIC at 25 μg/mL [19]. The mechanism of action of compound **1** involves membrane damage and inhibitory effects against ATP synthase in microorganisms. As a nonionic surfactant, compound **1** first approaches the binding site with its electronegative aldehyde oxygen atom, a potent hydrogen bond acceptor that will disrupt existing hydrogen bonds [19]. Recently, Montenegro et al. reported anti-phytopathogenic activity in compound **1** against two tomato plant bacteria pathogens, *Clavibacter michiganensis* subsp. *michiganensis* and *Pseudomonas syringae* pv. *tomato* [20]. Recently, it has been reported that polygodial affects the growth of normal and resistant isolates of *Botrytis cinerea* with EC_50_ values ranging between 117 and 175 ppm [21].

The aim of this work is to evaluate the antibacterial, antifungal, and anti-oomycete activity in microorganisms resistant to treatments used both in the clinic (ciprofloxacin, meropenem and fluconazole) and in fish farms (bronopol).

## 2. Results

### 2.1. Chemistry

**Figure 1:**
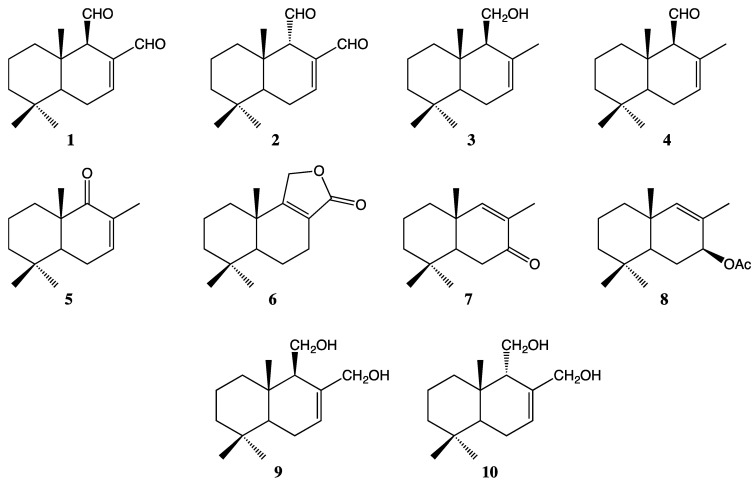
The chemical structures of drimanic compounds **1**–**10** used to evaluate against microorganisms.

Drimane compounds **1**–**10** (Figure 1) were obtained and characterized as described previously [3]. Detailed characterization can be found in Supplementary Materials (Schemes S1 and S2).

### 2.2. Antibacterial Activity

**Figure 2:**
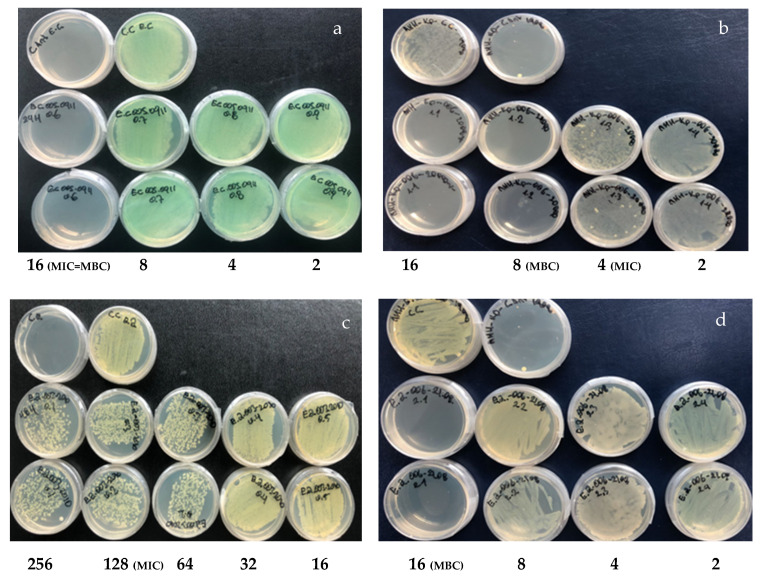
Antibacterial effect of drimanic compounds. Assays were performed on Müeller–Hinton agar plates. (**a**) Compound **1** assay against *P. aeruginosa* MIC and MBC at 16 µg/mL. (**b**) Compound **2** assay against *K. pneumoniae* MIC 8 µg/mL and MBC 16 µg/mL. (**c**) Compound **5** assay against *E. avium* MIC 128 µg/mL and MBC at 256 µg/mL. (**d**) Compound **7** assay against *E. avium* MIC and MBC 16 µg/mL.

The results of the MIC and MBC are presented in Figure 2 and Table 1. Of the ten molecules under study, polygodial (**1**) showed the most potent antibacterial activity compared to the other tested molecules; compounds **2** and **4** exhibited good activity; the remaining compounds (**5**–**8**) showed moderate activity; and compounds **9** and **10** were inactive against the tested strains.

### 2.3. Evaluation of Antifungal Activity

#### 2.3.1. Effect of Compounds **1**–**10** on Growth of Yeast

**Figure 3:**
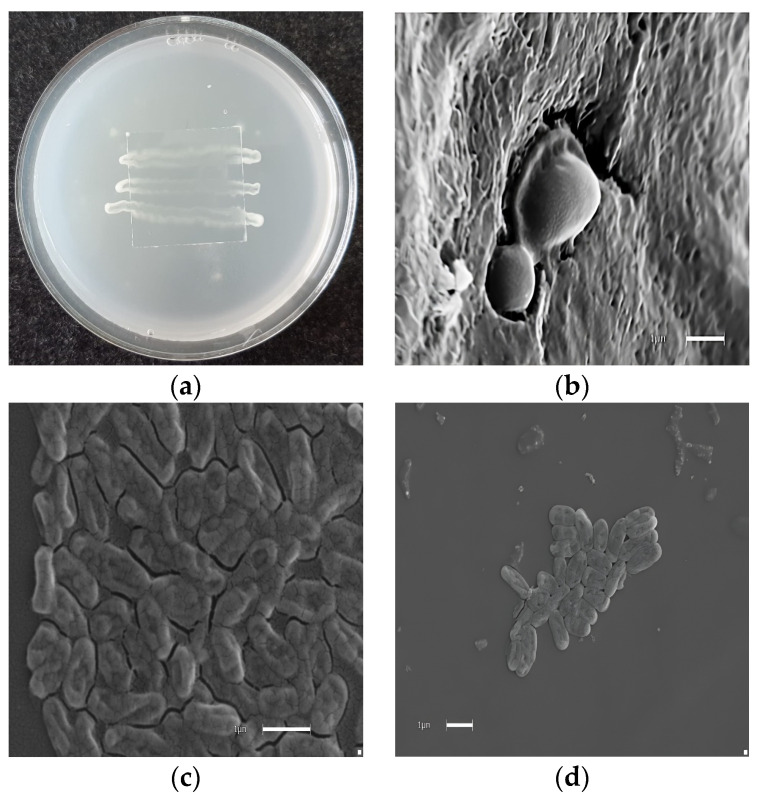
Morphological effect of polygodial on yeast. (**a**) Microculture in rice agar. (**b**) *Candida albicans* (control). (**c**) Microphotographs correspond to *C. albicans* growth in the presence of 16 µg/mL of polygodial (**1**) at 6 h post incubation. (**d**) Microphotographs correspond to *C. parasilopsis* growth in the presence of 16 µg/mL of polygodial. The final solvent concentration was identical in the control and treatment assays. Each bar corresponds to 1 µm.

For the best evaluation of the distinctive behavior of these compounds against yeasts, we selected the clinically significant strains *Candida albicans* as targets. *C. lusitanae*, *C. tropicalis*, *C. krusei*, and *C. glabrata* present a major clinical complication that threatens the lives of immunocompromised patients, especially those who have received solid organ transplantation [22]. Thus, the activity of drimanic compounds and their derivatives against this fungus is clinically relevant. On the other hand, *C. albicans* is a major cause of nosocomial bloodstream infections worldwide [22]. In this pathway, compounds **1**–**10**, which showed activity against at least one yeast, were tested against yeast strains by determining, this time, the MIC_80_ (the minimum concentration of compounds that inhibit 80% of growth), a less stringent endpoint widely used [23] and recommended by CLSI [24]. MIC_80s_ consistently represent compound’s in vitro activity and often correlate better with other antifungal activity measures [22]. The effect of compounds **1**–**10** on the growth of yeasts has also been assessed; the results are shown in Table 2. MIC and MFC studies were performed at different concentrations of these compounds, and the anti-yeast effect was quantified. The data indicate that aldehyde drimanic compounds (**1**, **2**, and **4**) inhibit growth in the culture medium of *Candida* species and strongly reduce in the presence of **1**, and this effect increases with exposition time and **1** concentration. After six hours of incubation, the percentage of membrane damage increases from 100% (Figure 3).

#### 2.3.2. Effect of Compounds **1**–**10** on the Growth of Filamentous Fungi

**Figure 4:**
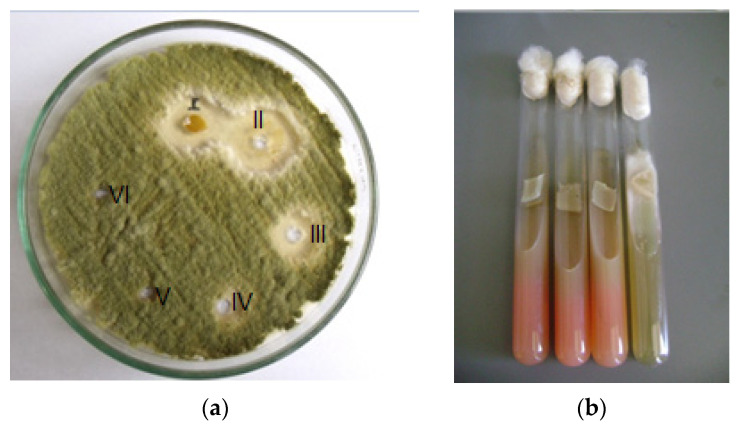
(**a**) *Aspergillus flavus* treated with polygodial: well I bark extract *D. winteri* 32 (µg/mL), well II polygodial 16 (µg/mL), well III polygodial 8 (µg/mL), well IV polygodial 4 (µg/mL), well V = 2 µg/mL, and well VI = 1 µg/mL; (**b**) control positive of *Fusarium oxisporum* and culture treated with 32 μg/mL of polygodial.

The results indicate that drimanic compounds can affect the growth of the filamentous fungi hyalohyphomycetes (*A. flavus*, *A. niger*, *A. terreus*, *A. fumigatus*, *F. solani*, and *F. oxisporum*) (Table 3). Figure 4 shows the percentage of inhibition of *A. flavus* at different polygodial concentrations. On the other hand, the results indicate that polygodial reduces the *F. oxysporum* mycelium growth by nearly 100% after 72 h of incubation at 32 μg/mL.

### 2.4. Anti-Oomycete Activity

**Figure 5:**
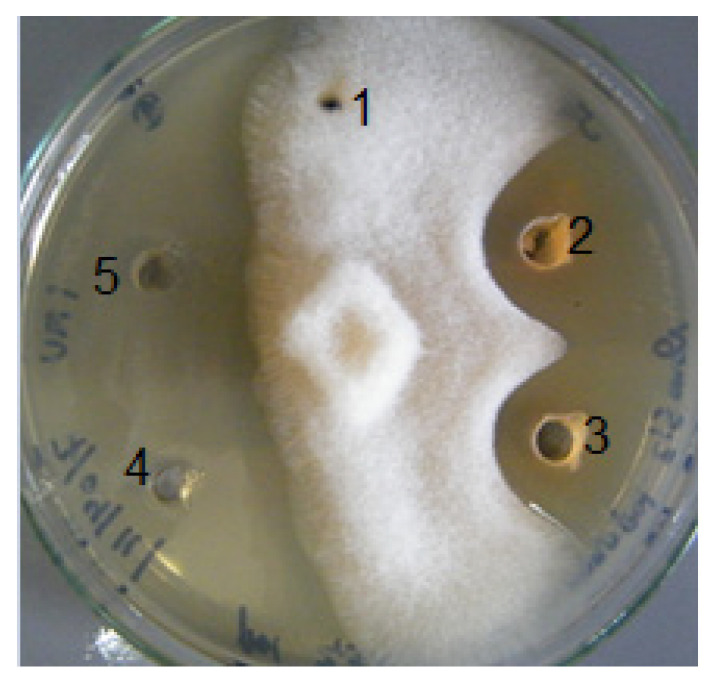
Anti-*saprolegnia* assay: wells 1 (control), 2, and 3 (compound **1** and compound **2**, respectively); 4 (bronopol) and 5 (fluconazole) positive controls.

The ability of drimanic compounds **1**–**10** to inhibit the growth of *Saprolegnia parasitica* and *Phytophthora cinnamomi* was studied. The minimum inhibitory concentrations (MICs) and the minimum oomyceticidal concentrations (MOCs) were determined by the microdilution method [24] using bronopol and fluconazole as positive controls (Figure 5 and Table 4), and the percentage of the membrane damage values for the compounds are summarized in Table 4.

## 3. Discussion

Previous work reports indicate that polygodial **1** has moderate antibacterial activity against Gram-positive bacteria, including *B. subtilis* and *S. aureus*, and Gram-negative bacteria, including *S. choleraesuis*, with an MIC of 100 μg/mL [18,25,26]. The present study showed that **1** has excellent antibacterial activity against *K. pneumoniae*, *E. avium*, *E. coli*, and *S. tiphy* with MBC values of 32 μg/mL, 16 μg/mL, 4 μg/mL, and 32 μg/mL and MIC values of 16 μg/mL, 8 μg/mL, 2 μg/mL, 16 μg/mL, and 4 μg/mL, respectively, which is consistent with the results obtained from MIC values of 7.8 μg/mL and 31.25 μg/mL for Gram-positive and -negative bacteria, respectively [26]. The high potency observed is attributable to the presence of an aldehyde group in position C8–C9 [27] and to the formation of pyrrolic derivatives with molecules that have a primary amino group [18,28]. However, the antibacterial activity of terpenes is determined by their functional group [29]; an oxygenated functional group has better antibacterial activity than hydrocarbons [30]. Although the mechanism of action of terpenes is not well-known, Griffin et al. reported that terpenes can inhibit two crucial processes that are essential for microbial survival, which includes oxygen consumption and oxidative phosphorylation [31].

Compound **3** has an MBC of >256 μg/mL and MIC of 256 μg/mL of treatment against *K. pneumoniae*, compound **4** has an MBC of 32 μg/mL and MIC of 16 μg/mL against *K. pneumoniae*; and MBC of 64 μg/mL and MIC of 64 μg/mL against *E. avium*. On the other hand, compound **5** has an MBC against *K. pneumoniae* of 64 μg/mL and MIC of 32 μg/mL and *E. avium* MBC of 64 μg/mL and MIC of 64 μg/mL against *P. aeruginosa*. Nevertheless, for compound **6,** our obtained result is much more relevant than that reported by Duraipandiyan MIC > 550 μg/mL [32]. Compounds **9** and **10** show no evidence of antibacterial activity to compare our results. Another relevant point of the antibacterial activity is the mechanism of action, which can vary with the type of terpene and/or the strain of microorganism used [33].

Anti-yeast studies were performed on the activity of the series **1**–**10** against *C. albicans* as targets. *C. lusitanae*, *C. tropicalis*, *C. krusei*, *C. glabrata*, and *C. parasilopsis* show significant differences. Thus, compounds **1**–**9** showed anti-yeast activity against at least one yeast and were retested against the standardized strains. In this opportunity, MIC_80_ (the minimum concentration of compounds inhibiting 80% of growth) was used, a less stringent endpoint widely used [23] and recommended by CLSI [24]. MIC_80_ consistently represents the in vitro activity of compounds and often provides a better correlation with other measures of antifungal activity [22]. The results are shown in Table 2. Since high differences were observed in the MFC between the compounds studied against the different yeasts, studies of damage against *C. albicans* of the compound, the most potent in the activity as an antifungal, were conducted and determined by electron microscopy effect at the membrane level and loss of normal morphology (Figure 3).

Regarding the antifungal activity against filamentous fungi, the sensitivity of hyalohyphomycetes to drimanes is evidenced. Therefore, it can be stated that (i) all compounds, except **9** and **10**, showed significant activity (MIC 8–128 µg/mL) against fungi of the genus *Aspergillus*, and are the most common species causing disease in primarily immunocompromised patients; (ii) five compounds of the series were active against *Aspergillus* and *Fusarium*; and (iii) all active compounds showed fungicidal rather than fungistatic properties against most fungi, as they not only inhibit but also kill them at MFC < 256 µg/mL. On the other hand, previous studies on phytopathogenic fungi reported by Carrasco et al. indicate that compound **1** reduces germination of *B. cinerea*, and this effect occurs mainly in the early stages of germination [21]. The results shown in Table 3 indicate that in the presence of 16 µg/mL, there is no formation of *A. fumigatus*. This filamentous fungus is the most frequent cause of invasive lung disease. Although the MCF of **1** is higher than the amphotericin B control, this polyene-type antifungal presents several ADRs (adverse drug reactions), such as nephrotoxicity, hepatotoxicity, hematological, and neurological alterations. Furthermore, in this study, we report activity against *Aspergillus* in comparison to that reported in [15]. In 2018, our research group reported activity against tomato phytopathogenic fungi that cause damage to agriculture [20]. The antimycotic effect of drimanic compounds is far superior to that observed with other antifungal agents [34,35,36]. For example, after 24 h of incubation from 1 to 8 µg/mL the growth of *Aspergilllus flavus* decreases, while under the same conditions, compound **2** decreases growth at 64 µg/mL (see Table 3).

The results of anti-oomycete activity showed that **1** and **2** were the most active compounds compared to the controls against the two strains tested. However, the MIC values of compound **8** for growth inhibition of *S. parasitica* and *P. cinnamomi* were 50 and 75 µg/mL, respectively, while for compound **9,** which differs by hydroxyl group with **1**, these values decreased to 75 µg/mL for the two strains, respectively. These data confirm what has been suggested in previous studies about the importance of the relationship between aldehydic and alcohol compounds and anti-oomycete activity [37,38]. It also confirms that aldehydic compounds (**1**–**2**) and compound **4** have a similar structure, where the anti-oomycete activity decreases in the tested microorganisms due to the absence of an α,β-unsaturated carbonyl system [38].

The nordrimanic compounds **5** and **7** with ketone group α,β-unsaturated carbonyl system exhibited moderate anti-Saprolegnia activity at concentrations (125–150 µg/mL), and only compound **6** showed low activity with similar effect to positive control fluconazole and bronopol against the two oomycetes tested. This decrease in the anti-oomycete activity of the drimane compounds could be related to the higher reactivity of the aldehyde groups over the keton groups and over the lactone compound **6,** leading to a decrease in the anti-Saprolegnia activity of **6** as occurs with other fungal microorganisms in this same assay [27,38]. In order to establish the possible route of death of the anti-oomycete strains, the membrane damage experiment was therefore carried out. This test involved testing the effect of the compounds against 2% caotropic agent (SDS), an anionic surfactant that causes 100% cell damage. The values of the percentage of membrane lysis of the oomycete species are summarized in Table 4. This type of assay is based on the direct action of the components on sterol formation in the oomycete membrane cells. In this respect, compound **1** caused the most damage to the membrane of the two oomycete strains tested, followed by compound bronopol and compound **2**. As in the previous case, compound **4** caused only 75 and 65% damage to *Sp* and *Pc*, respectively. The membrane damage exerted by compounds **1** and **2** corroborates the importance of the aldehydic group in the drimane structure in increasing the anti-oomycetic activity due to the high reactivity and the possibility of forming Michael-type reactions [15]. It has also been reported that the electronic distribution in the vicinity of double-bond 7,8 is important for activity, and other studies also found that the most active compound **1** possessed a larger differential positive region in the molecular electrostatic potential (MEP) major than **2**, which could contribute to the high antifungal activity of these molecules [15]. As for the absolute C9 configuration, here we found that compounds with an aldehyde in either of the two possible configurations did possess anti-oomycetic activity against *Sp* and *Pc*, although compound **2** shows two and four times less activity than **1** [15] (Table 4). Regarding the influence of the differential hydrophobicity of the drimanic compounds on the antifungal properties, even though these values indicate that they are all lipophilic *LogP* compounds, they present similar values for drimanes with clearly contrasting activity, suggesting that this parameter would not have a direct correlation with the antimycotic effect (Table 4). This report of anti-Saprolegnia activity of compounds **1** and **2** could be a solution to one of the major problems in salmon farming when treating saprolegniasis with formalin or bronopol by avoiding the side effects caused by these disinfectants.

## 4. Materials and Methods

### 4.1. Chemicals and Reagents

Reagents were purchased from Sigma Chemical Co. Inc. (St. Louis, MO, USA). All solvents were HPLC grade and were purchased from Merck (Darmstadt, Germany) and Fisher Scientific (New Jersey, NJ, USA). Müeller–Hinton broth was purchased from HiMedia Laboratories (Thane West, India).

### 4.2. Plant Extraction and Isolation

*D. winteri* bark collection, extraction, and isolation were performed as reported in our previous study [3].

### 4.3. Isolation of Natural Compounds and Preparation of Derivatives

The sesquiterpenes **1**–**4** (Figure 1) were isolated from dichloromethane extract of *D. winteri* bark. The extraction methodology, isolation, and identification of pure compounds were performed according to reported procedures [3]. Compounds **5**–**10** (Figure 1) were synthesized and identified using different protocols reported in the literature [3].

### 4.4. Antibacterial Activity

#### 4.4.1. Microbial Culture

For the antibacterial evaluation, standardized strains from the clinical microorganisms of Instituto de Microbiología clínica Universidad Austral de Chile were used in a first instance screening (*E. coli* EC-IMCL-15, *S. tiphy* SI-IMCL-23a, *P. aeruginosa* PA-IMCL-1-b, *K. pneumoniae* KP-IMCL-3b, and *E. avium* EA-IMCL-5b). The microorganism strains were kept at 4 °C until use. Standard microorganisms were subcultured on Müeller–Hinton broth. In addition, a suspension of each microbial culture was prepared in sterile Müeller–Hinton broth at a concentration of 0.5 McFarland.

#### 4.4.2. Determination of the Minimum Inhibitory Concentration (MIC)

Müeller–Hinton broth was distributed in 96-well microdilution plates. The sesquiterpene drimanes were then added to obtain a final concentration ranging from 256 to 0.125 μg/mL. The ATCC strains were mixed with Müeller–Hinton broth and standardized according to the turbidity of the 0.5 McFarland standard. The cell count was confirmed by spectrophotometry at 595 nm, and standardized bacterial suspensions were then added to the plates to obtain a final concentration of 10^5^ CFU/mL. The plates were incubated at 35 ± 2 °C for 24 h. The MIC was determined to be the lowest concentration capable of inhibiting bacterial growth. Bacterial growth was assessed through spectrophotometric analysis at a wavelength of 595 nm [39]. Negative control (culture medium) and growth control (culture medium and microorganisms) were used as standards.

#### 4.4.3. Determination of the Minimum Bactericidal Concentration (MBC)

Müeller–Hinton broth (HiMedia^®^) was distributed in Petri plates. The ATCC strains were mixed with Müeller–Hinton broth and standardized according to the turbidity of the 0.5 McFarland standard. The cell count was confirmed by spectrophotometry at 595 nm, and standardized bacterial suspensions were then added to the plates to obtain a final concentration of 10^5^ CFU/mL. The MBC was assessed by transferring bacterial suspensions with the sesquiterpene drimane from 256 μg/mL to 0.125 μg/mL and incubating at 35 ± 2 °C for 24 h and 48 h [39]. MBC was defined as the lowest concentration at which bacterial growth was not observed. Two independent experiments were conducted in duplicate.

### 4.5. Antifungal Evaluation

#### 4.5.1. Microorganisms and Media

For the antifungal evaluation, standardized strains from the American Type Culture Collection (ATCC), Rockville, MD, USA, and clinical microorganisms of Instituto de Microbiología clínica Universidad Austral de Chile were used in a first instance screening: *Candida albicans* ATCC 10231 as targets. Clinical strains: *C. lusitanae* CL-IMCL-1, *C. tropicalis* CT-IMCL-3, *C. krusei* CK-IMCL-4, *C. glabrata* CG-IMCL-6, CP-IMCL-P1, and *C. parasilopsis* hyalohyphomycetes (*A. flavus* AF-IMCL-3b, *A. niger* AN-IMCL-2a, *A. terreus* AN-IMCL-4, *A. fumigatus* ATTC 26934, *F. solani* FS-IMCL-2, and *F. oxisporum* FO-IMCL-2a).

Strains were grown on Sabouraud chloramphenicol agar slants for 48 h at 30 °C, maintained on slopes of Sabouraud dextrose agar (SDA, Oxoid, Hampshire, UK), and subcultured every 15 days to prevent pleomorphic transformations. Inocula of cells or spore suspensions were obtained according to reported procedures and adjusted to 1–5 × 10^3^ cells/spores with colony forming unit (CFU)/mL [40,41].

#### 4.5.2. Antifungal Susceptibility Testing Minimum Inhibitory Concentration (MIC)

The MIC of each compound was determined by using broth microdilution techniques according to the guidelines of the CLSI for yeasts (M27-A3) and for filamentous fungi (M 38 A2) [40,41]. MIC values were determined in RPMI-1640 (Sigma, St. Louis, MO, USA) buffered to pH 7.0 with MOPS. Microtiter trays were incubated at 35 °C for yeasts and hialohyphomycetes and at 28–30 °C, and MICs were visually recorded at 48 h for yeasts and at a time according to the control fungus growth for the rest of fungi.

For the assay, stock solutions of pure compounds were two-fold diluted with RPMI from 2 to 0.98 µg/mL (final volume = 100 µL) and a final DMSO concentration ≤ 1%. A volume of 100 µL of inoculum suspension was added to each well with the exception of the sterility control, where sterile water was added to the well instead. Ketoconazole, terbinafine, and amphotericin B were used as positive controls.

Endpoints were defined as the lowest drug concentration resulting in total inhibition (MIC80) of visual growth compared to the control wells containing no antifungal. MIC_80_ was defined as the lowest concentration of a compound that inhibited 50% of the growth control, respectively (culture media with the microorganism but without the addition of any compound), and was determined spectrophotometrically with the aid of a VERSA Max microplate reader (Molecular Devices, Sunnyvale, CA, USA).

The minimum fungicidal concentration (MFC) of each compound against each strain was also determined as follows: After determining the MIC_80_, an aliquot of 5 µL sample was withdrawn from each clear well of the microtiter tray and plated onto a 150 mm RPMI-1640 agar plate buffered with MOPS (Remel, Lenexa, KS, USA). Inoculated plates were incubated at 30 °C, and MFCs were recorded after 48 h. The MFC was defined as the lowest concentration of each compound that resulted in total inhibition of visible growth.

#### 4.5.3. Determination of MIC and MOC

Compounds for which mycelium presence was recorded as negative at the concentration of 250 µg/mL were tested for MIC values ranging from 12.5 to 250 µg/mL using the above method [42]. Control plates were treated with bronopol and fluconazole. MIC was read visually at 72 h and was defined as the concentration of compounds that inhibited growth by at least 80% or more relative to growth control. MOC was defined as the lowest concentration of the chemicals that prevented visible growth or germination of mycelium.

#### 4.5.4. Membrane Damage

Saprolegnia strains 113-IMCL and *Phytophthora cinnamomi* PC-LFPL-1 were cultured by shaking at 20 °C and then washed twice and diluted to approximately 3 × 10^4^ zoospores/mL with cold MOPS buffer, pH 6.0. Cells were aliquoted to tubes, and **1**–**10** was added at a final concentration of 150 µg/mL. SDS (2%) was used as reference compound, which produces 100% cellular oomycete leakage. Saprolegnia were incubated at 20 °C, and samples were taken at time intervals (6, 12, 24, and 48 h) and spun at 3500 rpm for 7 min in microcentrifuge tubes. The supernatants were collected for absorbance analysis at 260 nm in a Beckman DU-600 spectrophotometer (Brea, CA, USA) [43]. Results were the means of values from at least two independent assays.

### 4.6. Statistical Analyses

All experiments were performed in triplicate. Descriptive statistical analyses were performed using GraphPad Prism 9 for Mac. Statistical significance between control sets and treated groups was analyzed using a two-way analysis of variance (ANOVA) followed by Tukey’s multiple comparison tests. *p* values < 0.05 were considered significant.

## 5. Conclusions

The present study showed that most of the microorganisms were susceptible to the drimanic compounds compared to the tested antibiotics, antifungals, and chemicals that usually cause serious health and environmental problems. Drimanic compounds cause different bioactivities depending on the functional group contained in each structure. In the case of polygodial, the aldehyde systems play a determining role, and this correlates with its antifungal, antibacterial, and anti-oomycete activity.

This work is the first report of natural and hemisynthetic drimane compounds against *Saprolegnia parasitica*.

## Figures and Tables

**Table 1:** MIC and MBC (µg/mL) values of the drimane series against nosocomial bacteria.

Compound	*K. pneumonia*		*E. avium*		*E. coli*		*P. aeruginosa*		*S. tiphy*	
	MIC	MBC	MIC	MBC	MIC	MBC	MIC	MBC	MIC	MBC
**1**	16	32	8	16	2	4	16	32	4	8
**2**	64	128	16	32	16	32	32	64	4	16
**3**	256	>256	256	256	256	256	>256	>256	128	256
**4**	16	32	64	64	32	32	64	128	64	64
**5**	32	64	64	64	64	64	128	128	128	256
**6**	16	32	64	64	128	256	256	256	128	128
**7**	32	64	128	128	32	64	64	128	128	256
**8**	64	128	64	128	64	128	128	>256	128	>256
**9**	256	>256	256	256	>256	>256	>256	>256	>256	>256
**10**	>256	>256	>256	>256	>256	>256	>256	>256	>256	>256
**Doripenem**	128	256	128	256	256	256	>256	>256	>256	>256
**Ciprofloxacin**	>256	>256	>256	>256	>256	>256	>256	>256	>256	>256

**Table 2:** MIC and MFC (µg/mL) values of representative members of the drimane series **1**–**10** against *Candida* strains.

Compound	*C. albicans*		*C. lusitaneae*		*C. tropicalis*		*C. krusei*		*C. glabrata*		*C. parasilopsis*	
	MIC	MFC	MIC	MFC	MIC	MFC	MIC	MFC	MIC	MFC	MIC	MFC
**1**	8	16	8	8	32	32	16	16	64	64	8	32
**2**	64	128	64	64	64	64	128	128	128	128	32	64
**3**	128	128	128	128	64	64	64	64	256	256	64	128
**4**	32	64	16	32	64	128	32	64	128	256	32	32
**5**	64	64	256	256	256	256	64	64	32	32	64	128
**6**	128	256	128	128	128	256	128	>256	128	>256	128	>256
**7**	64	128	32	64	128	64	128	64	128	256	128	256
**8**	64	128	64	128	64	128	32	64	32	64	16	32
**9**	128	>256	128	>256	>256	>256	>256	>256	128	>256	>256	>256
**10**	>256	>256	>256	>256	>256	>256	>256	>256	>256	>256	>256	>256
**Terbinafine**	0.125	0.125	0.250	0.250	1	1	1	1	4	8	4	8
**Fluconazole**	1	1	0.250	0.250	0.5	0.5	0.5	0.5	2	0.5	2	4

**Table 3:** MIC and MFC (µg/mL) values of representative members of the drimane series **1**–**10** against filamentous fungi.

Compound	*A. flavus*		*A. niger*		*A. terreus*		*A. fumigatus*		*F. solani*		*F. oxisporum*	
	MIC	MFC	MIC	MFC	MIC	MFC	MIC	MFC	MIC	MFC	MIC	MFC
**1**	8	16	8	8	32	32	16	16	64	64	8	32
**2**	64	128	64	64	64	64	128	128	128	128	32	64
**3**	128	128	128	128	64	64	64	64	256	256	64	128
**4**	32	64	16	32	32	64	16	256	256	256	32	32
**5**	64	64	256	256	256	256	64	64	32	32	64	128
**6**	128	256	128	64	128	256	128	>256	128	>256	128	>256
**7**	64	128	128	128	64	128	64	128	32	64	32	64
**8**	64	256	128	128	128	128	128	128	128	128	128	256
**9**	128	256	128	>256	128	>256	128	>256	128	>256	128	>256
**10**	128	128	128	256	128	256	128	256	128	128	128	256
**AmfoB**	0.125	0.125	0.250	0.250	1	1	1	1	2	2	4	8
**Itraconazole**	1	1	0.250	0.250	0.5	0.5	0.5	0.5	2	0.5	2	4

**Table 4:** In vitro anti-oomycete activity values (µg/mL) of drimanic compounds against *S. parasitica* and *P. cinnamomi*.

Compound	*Log P*	MIC ^a^ *Sp*	MIC ^a^ *Pc*	MOC ^a^ *Sp*	MOC ^a^ *Pc*	Damage (%) ^b^	
						*Sp*	*Pc*
**1**	2.33	12.5	12.5	12.5	12.5	100	100
**2**	2.33	12.5	25	25	50	100	100
**3**	3.83	200	>200	200	>200	0	0
**4**	3.53	75	100	75	100	75	65
**5**	3.84	125	150	125	150	25	20
**6**	3.35	>200	>200	>200	>200	0	0
**7**	3.37	100	125	150	150	20	15
**8**	4.65	50	50	50	50	75	50
**9**	2.77	75	100	75	100	25	30
**10**	2.77	150	175	150	175	20	20
**Bronopol**		>200	>200	>200	>200	30	30
**Fluconazole**		150	175	150	175	Nd	Nd
**SDS**		Nd	Nd	Nd	Nd	100	100

Nd: Not determined; ^a^ each value represents the mean SD of three experiments, performed in triplicate; and ^b^ damage produced by compounds **1**–**8** compared to the damage produced by sodium dodecyl sulfate (SDS). SDS was utilized at a final concentration of 2% that produced 100% of cell lysis. The assay was performed in duplicates.

## Data Availability

Any unpublished raw data associated with this research are available by contacting the corresponding author I.M.

## Supplementary Materials

The following supporting information can be downloaded at: https://www.mdpi.com/article/10.3390/molecules29122844/s1. Schemes S1 and S2: Conditions and Reagents for synthesis of compounds **2**, **4**–**10**. Chemical characterization of compounds **2**, **4**, **5**, **7**–**10**.
