# Supplementary material for: Antimicrobial Activity of Drimanic Sesquiterpene Compounds from Drimys winteri against Multiresistant Microorganisms
Source: Molecules. 2024 Jun 14;29(12):2844. doi: 10.3390/molecules29122844 (PMC11206827; doi:10.3390/molecules29122844)
Supplement: Supplementary file 1 [file molecules-29-02844-s001.zip › molecules-2961934-supplementary.pdf]

SUPPORTING INFORMATION

Antimicrobial Activity of Drimanic Sesquiterpene Compounds from *Drimys winteri* against Multiresistant Microorganisms

Iván Montenegro [1,*], Rolando Pazmiño [2], Ileana Araque [2], Alejandro Madrid [3], Ximena Besoain [4], Enrique Werner [5], Luis Espinoza-Catalán [6], Andrés F. Olea [7], Claudio Parra [8], Valentina Navarrete Molina [1], Patricio Godoy [9], Yusser Olguín [10,11,12] and Mauricio A. Cuellar [2,*]

* Correspondence: ivan.montenegro@uv.cl (I.M.); mauricio.cuellar@uv.cl (M.A.C.); Tel.: +56-32-2508439 (M.A.C.)

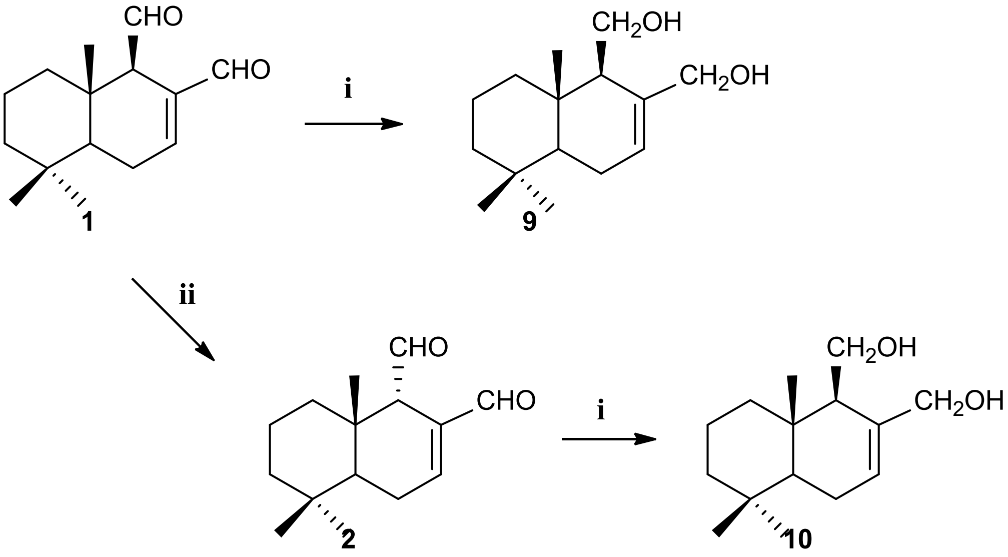

Scheme S1: *Conditions and reagents*: (i) $NaBH_4$, MeOH, r.t, 2 h **9** **(**62%); (ii) NaOH, MeOH, r.t, 20 min;**2** (92%) (i) $NaBH_4$, MeOH, r.t, 2 h **10** (48%)

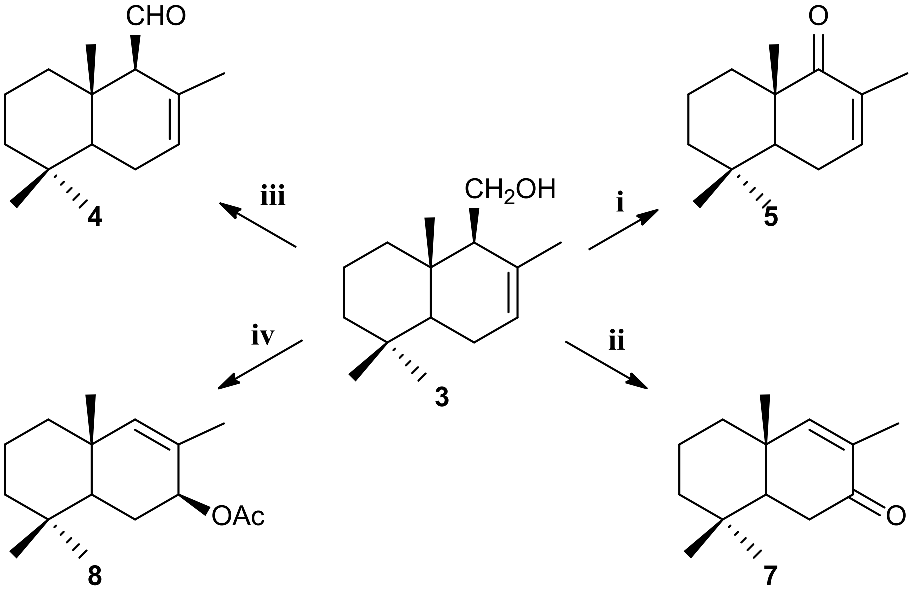

Scheme S2: *Conditions and reagents*: (i) Jones' reagent, 0 °C, 1 h, **8**; 60%, **5** (ii): Pyridinium chlorochromate PCC, CH_2Cl_2, N_2, r.t, 2 h, **7**; 60%.; (iii) PCC, CH_2Cl_2, 0 °C, 1 h **4** (73%); (iv) $Pb(OAc)_4$, benzene, reflux, **7**; (60%).

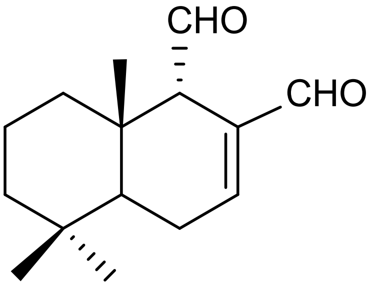

2

Polygodial (**1**) (2.0 mmol) was dissolved in MeOH (30 mL) containing sodium methoxide. After two hours of stirring the reaction mixture was washed with water and extracted with hexane. The organic phase was dried, concentrated and purified by column chromatography yielding compound **2** (1.83 mmol), known as isopolygodial or epi-polygodial, in 92 % yield. [◎◎$_D^{25}$: +30º (c = 1,00 $CHCl_3$). IR (KBr): 2927, 2850, 2726, 1722, 1680, 1642, cm^{-1} ^{1}H NMR (400 MHz, $CDCl_3$, δ, ppm): 9.85 (1H, dd, *J* = 11.7 Hz, H-11); 9.40 (1H, s, H-12); 7.10 (1H, m, H-7), 3.25 (1H, s, H-9); 0.98 (3H, s, Me-15); 0.90 (3H, s, Me-13); 0.89 (3H, s, Me-14). ^{13}C NMR (100 MHz, $CDCl_3$, δ, ppm): 202 (C-11); 193 (C-12); 153.6 (C-7) 137.5 (C-8); 58.10 (C-9); 44.2 (C-5); 42.0 (C-3); 37.5 (C-10), 37.0 (C-1); 33.0 (C-4); 32.8 (C-13); 25.2 (C-6); 22.0 (C-14); 18.0 (C-2); 15.0 (C-15); MS: *m*/*z*: 234.

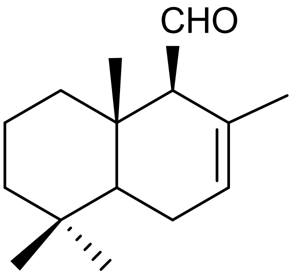

4

225 mg of drimenol (**3**) (1.13 mmol) was diluted in 100 mL DCM and stirred at room temperature. Subsequently PDC (1010.6 mg, 2.63 mmol) was added. After 3 hours the mixture was filtered through silica gel with AcOEt, the organic phase was dried over anhydrous Na_2SO_4 and concentrated under reduced pressure. The resulting crude was purified by column chromatography to give a colorless drimenal oil (**4**), (162.7 mg, 7.4 mmol) in 73% yield. $[\alpha]_D^{25}$ −20 º ($CHCl_3$, c = 1.0); IR (film) ◎$_{max}$ 2923, 2851, 1714 cm^{-1}. ^{1}H NMR (400 MHz, $CDCl_3$, δ, ppm): 9.69 (1H, d, *J* = 5.1 Hz, H-11); 5.70 (1H, bs, H-7); 2.59 (1H, m, H-9); 2,05 (2H, m, H -6α and β), 1.70-

1.10 (7H, m, H-1α and β, H-2α y β, H-3α and β, H-5), 1.62 (3H, s, Me-12), 1.05 (3H, s, Me-15), 0.90 and 0.85 (6H, 2s, Me-13 and Me-14). ^{13}C NMR (100 MHz, $CDCl_3$, δ, ppm): 206.7 (C-11); 127.8 (C-8); 125.5 (C-7); 67.6 (C-9); 49.1 (C-5); 42.0 (C-3); 40.4 (C - 1); 37.0 (C-10); 33.3 (C-14); 33.0 (C-4); 23.7 (C-6); 22.1 (C-13); 21.6 (C-12); 18.3 (C-2); 15.7 (C-15). EM *m*/*z*: 220 [M +].

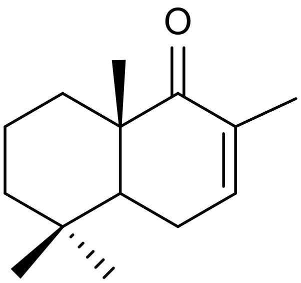

5

Compound **3** (1.0 g, 4.85 mmol) was dissolved in acetone p.a. (50 mL), cooled to 0 °C and under stirring a solution containing Jones' reagent was slowly added until the orange color persisted over time. The reaction mixture was stirred for one hour, then isopropyl alcohol was added to destroy the excess reagent. Water was added to the reaction mixture and extracted with AcOEt, the organic phase was dried with $MgSO_4$, concentrated at rotary evaporator, and purified by column chromatography yielding ketone **5** (0.6 g, 60%) as a white solid of mp: 77.0-77.7 °C; $[\alpha]_D^{25}$ −74 ° ($CHCl_3$, c = 1.0); IR (film) ν_{max} 2923, 1663 cm^{-1}. ^{1}H NMR (400 MHz, $CDCl_3$, δ, ppm): 6.69 - 6.67 (1H, m, H-7); 2.30-2.25 (2H, m, H-6α and β); 1,89 (1H, bd, *J* = 13.4 Hz, H-1); 1.75 (3H, d, *J* = 2.0 Hz, Me-12); 1.70-1.10 (6H, m, H-1α, H-2α and β, H 3α-and β, H-5); 1.04 (3H, s, Me-13); 0.99 and 0.91 (6H, 2s, Me-15 and 14). ^{13}C NMR (100 MHz, $CDCl_3$, δ, ppm): 205.9 (C-9); 143.4 (C-7); 132.9 (C-8); 49.4 (C-5); 45.1 (C-10); 41.6 (C-3); 33.6 (C-4), 33.2 (C-1); 32.3 (Me-14); 24.4 (C-6); 22.3 (Me-13); 18.2 (C-2); 17.1 (Me-15), 16.4 (Me-12). MS *m*/*z* (EI): 206 [M +1].

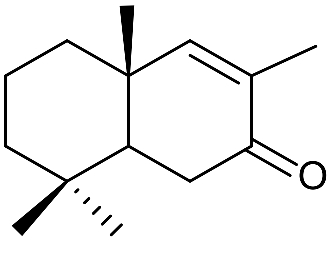

7

To a solution of compound **3** (10 mmol), PCC (10 mmol of the oxidant in DCM (50 ml) was added. A brown solution was obtained, which was stirred for 24 h until thin layer chromatographic analysis showed the disappearance of the starting material. Celite (500 mg) with (50 mL) AcOEt was added to the reaction mixture and the suspension was filtered through silica gel, washing thoroughly with AcOEt. The filtrate was dried with $MgSO_4$, concentrated, and purified by column chromatography yielding compound **7** (1.2 mmol) as a colorless oil in 60% yield; $[\alpha]_D^{25}$ −7 ° ($CHCl_3$, c = 1.0); IR (film) ν_{max} 2956, 1704, 1673 cm^{-1}. ^{1}H NMR (400 MHz, $CDCl_3$, δ, ppm): 6.39 (1H, d, *J* = 1.3 Hz, H-9); 2,49 (1H, dd, *J* = 3.9 and 17.4 Hz, H-6◎); 2.34 (1H, dd, *J* = 14.0 and 17.4 Hz, H-6β); 1.72 (3H, d, *J* = 1.3 Hz, Me-12); 1.72 (1H, dd, *J* = 13.9 and 3.9 Hz, H-1β); 1.70-1.10 (6H, m, H-1α, H-2α and β, H 3α-and β, H-5); 1.07 (3H, s, Me-15); 0.91 (3H, s, Me-13); 0.88 (3H, s, Me-14). ^{13}C NMR (100 MHz, $CDCl_3$, δ, ppm): ◎201.6 (C-7); 158.3 (C-9); 131.2 (C-8); 50.6 (C-5); 41.2 (C-3); 38.4 (C-4); 36.9 (C-10); 35.4 (C-1); 32.8 (C-6); 32.2 (Me-13); 20.9 (Me-14); 18.6 (C-2); 18.5 (Me-15); 15.5 (Me-12). MS *m*/*z*: 206 [M +].

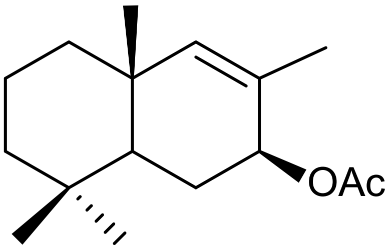

8

Compound **3** (3.6 g, 16.1 mmol) was dissolved in 50 mL benzene, then lead tetraacetate (LTA) was gradually added (7.2 g 16.2 mmol). The solution turned pale yellow and was left to reflux for 2 hours. The reaction was monitored by TLC until the starting material disappeared. A greyish white precipitate was separated by filtration. The filtrate was diluted with AcOEt and washed three times with water. The organic phase was treated with $NaHCO_3$, dried with $MgSO_4$, concentrated, and purified by column chromatography to give a yellow liquid compound **8** (3.5 g, 87%). (420 mg, 95%): IR (KBr): 1730, 1245 cm^{-1} (OAc); ^{1}H NMR (400 MHz, $CDCl_3$, δ, ppm): 5.40 (1H, m, H-9), 2.08 (3H,s, OAc), 1,55 (3H, s, Me-12),

0.98 (3H, s, Me-15), 0.87 (3H, s, Me-13), 0.82 (3H, s, Me-14), ^{13}C NMR (100 MHz, $CDCl_3$, δ, ppm): (<u>C</u>OCH_3 171.1); 141.1 (C-9); 128.6 (C-8); 74.8 (C-7); 49.7 (C-5); 41.7 (C-3) 39.2 (C-10); 35.7 (C-1); 32.8 (C-4); 32.7 (C-14); 25.8 (C-6); 21.3 (CH_3CO); 21.2 (C-13); 18.8 (C-2); 18.8 (C-12). MS *m*/*z*: 250 [M + 1].

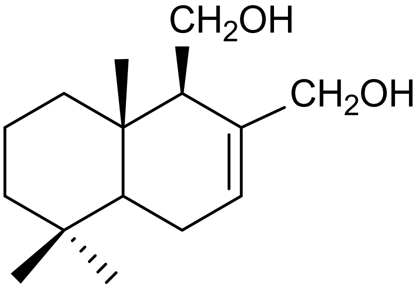

9

To a solution of compound **1** (2.1 mmol) in MeOH (25 mL), $NaBH_4$ (8.4 mmol) was carefully added in small portions. The mixture was heated under reflux at 50 °C for 4 h and most of the solvent was evaporated under vacuum. Water (20 mL) was added, acidified with 5 drops of 1 M HCl cautiously and extractions with AcOEt were performed. The organic phase was dried with MgSO4, evaporated, and subjected to column chromatography (CC) to give compound **9** (1.3 mmol) in 62% yield: Yellow solid; mp: 74 °C; $[\alpha]_D^{25}$ −8° ($CHCl_3$, *c* = 1.0). IR (film) ν_{max} 3286, 2924 cm^{-1}. ^1H NMR (400 MHz, $CDCl_3$, δ, ppm): 5.78 (1H, m, H-7); 4.33 (1H, d, *J* = 11.9 Hz, H-12 β); 4.00–3.90 (4H, m, H-11B, 12 *α* and 2OH); 3.64 (1H, dd, *J* = 8.7 and 10.4 Hz, H-11*α*); 2.15–1.90 (4H, m, H-1β, 6*α*, 6β and 9); 1.58–1.40 (3H, m, H-2*α*, 2β and 3β); 1.24–1.10 (3H, m, H-1*α*, 3*α* and 5); 0.88 and 0.86 (6H, 2s, Me-13 and Me-14); 0.75 (3H, *s*, 15-Me). ^{13}C NMR (100 MHz, $CDCl_3$, δ, ppm): 136.9 (C-8); 127.1 (C-7); 67.3 (C-12); 61.2 (C-11); 54.4 (C-5); 49.4 (C-9); 42.0 (C-3); 39.3 (C-1); 35.6 (C-10); 33.2 (C-13); 32.9 (C-4); 23.5 (C-6); 21.9 (C-14); 18.8 (C-2); 14.5 (C-15). MS *m*/*z* (EI): 238 [M^+].

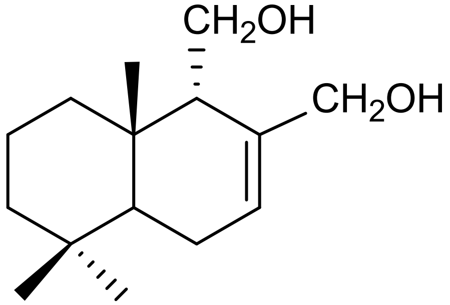

10

Polygodial **1** (2.5 g, 10.4 mmol) was dissolved in MeOH (100 mL) containing NaOH (5.0 g). The resulting solution was stirred for 20 min at room temperature to allow epimerization of C-9, then $NaBH_4$ (2.9 g, 14.96 mmol) was carefully and gradually added, after 4 hours some of the solvent was removed at rotary evaporator, water was added and extracted with AcOEt. It was concentrated and purified by column chromatography eluting with a hexane-AcOEt mixture in concentration gradient yielding two polar products: eluting the C-9α diol (1.20 g, 48%) as a white solid **10** at mp. 129.5-130 °C. $[\alpha]_D^{25}$ +10° ($CHCl_3$, c = 1.0). IR (film) υ_{max} 3286, 2924, 1640, 1045 cm^{-1}. ^{1}H NMR (400 MHz, $CDCl_3$, δ, ppm): 5.83 (1H, t, *J* = 3,5 Hz, H-7); 4.13 (1H, d, *J* = 12.0 Hz, H-12β); 4.00 (1H, d, *J* = 12.0 Hz, H-12 α); 3.92 (1H, dd, *J* = 4.5 and 11.0 Hz, H-11 β); 3.64 (1H, dd, *J* = 5.4 and 11.0 Hz, H-11α); 2.60 (2H, bs, 2OH); 2.20–1.90 (3H, m, H-6α and β, H-9β); 1.67–1.20 (7H, m, H-1α and β, H-2α and β, H-3α and β, H-5); 0.92 (6H, 2s, Me-15 and Me-14); 0.88 (3H, s, Me-13). ^{13}C NMR (100 MHz, $CDCl_3$, δ, ppm): 137.2 (C-8); 127.5 (C-7); 67.7 (C-12); 63.1 (C-11); 54.1 (C-5); 43.4 (C-9); 42.7 (C-3); 36.5 (C-1); 35.9 (C-10); 33.1 (C-4); 33.0 (C-15); 24.3 (C-6); 22.0 (C-14); 21.7 (C-13); 18.8 (C-2). MS *m/z* (EI): 238 [M + 1].
